# Molecular epidemiology of *Mycobacterium africanum* in Ghana

**DOI:** 10.1186/s12879-016-1725-6

**Published:** 2016-08-09

**Authors:** Adwoa Asante-Poku, Isaac Darko Otchere, Stephen Osei-Wusu, Esther Sarpong, Akosua Baddoo, Audrey Forson, Clement Laryea, Sonia Borrell, Frank Bonsu, Jan Hattendorf, Collins Ahorlu, Kwadwo A. Koram, Sebastien Gagneux, Dorothy Yeboah-Manu

**Affiliations:** 1 Noguchi Memorial Institute for Medical Research, University of Ghana, Legon, Ghana; 2 Department of Medical Parasitology and Infection Biology, Swiss Tropical and Public Health Institute, Basel, Switzerland; 3 University of Basel, Basel, Switzerland; 4 Department of Chest Diseases, Korle-Bu Teaching Hospital, Korle-bu, Accra, Ghana; 5 37 Military Hospital, Accra, Ghana; 6 National Tuberculosis Programme, Ghana health Service, Accra, Ghana; 7 Department of Epidemiology and Public Health, Swiss Tropical and Public Health Institute, Basel, Switzerland

## Abstract

**Background:**

*Mycobacterium africanum* comprises two phylogenetic lineages within the *M. tuberculosis* complex (MTBC) and is an important cause of human tuberculosis (TB) in West Africa. The reasons for this geographic restriction of *M. africanum* remain unclear. Here, we performed a prospective study to explore associations between the characteristics of TB patients and the MTBC lineages circulating in Ghana.

**Method:**

We genotyped 1,211 MTBC isolates recovered from pulmonary TB patients recruited between 2012 and 2014 using single nucleotide polymorphism typing and spoligotyping. Associations between patient and pathogen variables were assessed using univariate and multivariate logistic regression.

**Results:**

Of the 1,211 MTBC isolates analysed, 71.9 % (871) belonged to Lineage 4; 12.6 % (152) to Lineage 5 (also known as *M. africanum* West-Africa 1), 9.2 % (112) to Lineage 6 (also known as *M. africanum* West-Africa 2) and 0.6 % (7) to *Mycobacterium bovis*. Univariate analysis revealed that Lineage 6 strains were less likely to be isoniazid resistant compared to other strains (odds ratio = 0.25, 95 % confidence interval (CI): 0.05–0.77, *P* < 0.01). Multivariate analysis showed that Lineage 5 was significantly more common in patients from the Ewe ethnic group (adjusted odds ratio (adjOR): 2.79; 95 % CI: 1.47–5.29, *P* < 0.001) and Lineage 6 more likely to be found among HIV-co-infected TB patients (adjOR = 2.2; 95 % confidence interval (CI: 1.32–3.7, *P* < 0.001).

**Conclusion:**

Our findings confirm the importance of *M. africanum* in Ghana and highlight the need to differentiate between Lineage 5 and Lineage 6, as these lineages differ in associated patient variables.

**Electronic supplementary material:**

The online version of this article (doi:10.1186/s12879-016-1725-6) contains supplementary material, which is available to authorized users.

## Background

Tuberculosis (TB) remains one of the main global public health problems, particularly in resource-limited settings [1]. With an estimated 9 million new cases annually, and a pool of two billion latently infected individuals, control efforts are struggling in most parts of the world. The TB problem is further exacerbated by a strong synergy with Human immunodeficiency virus infection and acquired immune deficiency syndrome (HIV/AIDS), which is a particularly big challenge in sub-Saharan Africa. Moreover, the global emergence of drug resistance increasingly complicates TB control. The Word Health Organization (WHO) estimates that in 2013, of the 1.1 million TB cases co-infected with HIV, 80 % occurred in Africa, making Africa the hardest hit of the two epidemics [1].

Human TB is mainly caused by *Mycobacterium africanum* (MAF) and *Mycobacterium tuberculosis* sensu stricto (MTB), both members of *Mycobacterium tuberculo*sis complex (MTBC), which also includes several sub-species adapted to a variety of wild and domestic animals [2, 3]. MAF first identified in 1968 in Senegal, was initially described biochemically as an intermediary between MTB and *M. bovis* [4]. Like MTB, MAF strains were found to be sensitive to pyrazinamide; like *M. bovis*, they tended to be a weak producer of niacin, microaerophilic, and unable to reduce nitrate to nitrite [5]. Furthermore, similar to *M. bovis* they are unable to use glycerol as a sole carbon source due to the lack of functional pyruvate kinase [6]. Initial biochemical features subdivided MAF into two separate groups; the West-African sub-species and East-African also referred to as MAF type 1 and 2 [7]. However, based on genomic deletion analyses [2, 8, 9], we now know that MAF type 2 variant is a sub-lineage of MTB Lineage 4, which has been reclassified as MTB “Uganda sublineage” [10]. MAF type 1 or the “true” *M. africanum* consists of two phylogenetically distinct lineages that differ in their geographic distribution: Lineage 5 (also known as MAF West African 1) is found in the eastern part of West-Africa, and Lineage 6 (also known as West-Africa genotype II) is mainly found in the western part of West-Africa. A few countries like Ghana and Cote d’Ivoire harbor both Lineages 5 and 6 [9, 11].

The reason why MAF is largely restricted to the West African region remains unknown, but one possibility might be that MAF is adapted to distinct human population (s) in West Africa [9]. In support of this hypothesis, using a limited retrospective collection of MTBC isolates from South-western Ghana, we recently reported an association between MAF and the Ewe ethnic group [12]. One of the limitations of that previous study was that due to lack of relevant data, we could not control for possible confounding by HIV co-infection. Hence, we followed-up on this initial observation with a larger population-based prospective study in which we included more detailed clinical data and TB cases from both the southern and northern part of Ghana.

## Methods

### Ethical Statement and Patient inclusion criteria

The Institutional Review Board of the Noguchi Memorial Institute for Medical Research (NMIMR) and the Ethikkommission Beider Basel (EKBB) in Basel, Switzerland approved the study and its protocols. Following informed consent, consecutive sputum smear-positive TB cases were recruited from September 2012 to April 2014 from all TB diagnostic centres in the Accra Metropolitan (Southern) and the Mamprusi East (Northern Region) Health Administrations. The standard procedure for sputum sample collection as outlined by the National Tuberculosis Program (NTP) for routine diagnosis of TB in Ghana was used in this study. Participants provided written consent unless the participant was illiterate; in which case witnessed oral consent was used. In accordance with ethical review board regulation in Ghana consent was sought from guardians of children below the age of 18 before enrolment into the study and in some cases child assent was also sought. Samples were taken only after a detailed explanation of the study aims and written or thumb-printed consent obtained for participation. Only newly diagnosed smear-positive, pulmonary TB patients before initiation of treatment or less than 4 weeks of treatment were enrolled into the study. All eligible TB patients were encouraged to undergo HIV testing before initiation of anti-TB treatment according to national guidelines. Data collected from enrolled patients included age, sex, immunosuppressive co-morbidity with HIV, place of work, previous episode of TB, housing type, place of residence, ethnicity, smoking, level of education, income, and presence of Bacillus Calmette-Guerin (BCG) scar. All patients and staff involved with the study were blinded to the final data obtained and none of the authors have a conflict of interest in the study.

### Laboratory tests

The sputum samples were re-examined for the presence of acid-fast bacilli (AFBs) at NMIMR and cultured on solid agar, after which isolate deoxyribonucleic acid (DNA) was extracted for genotyping analysis. Briefly, we took a 10 μL loop full from a Lowenstein-Jensen medium slope and, after heat killing, extracted DNA first by digestion with lysozyme and proteinase K, solubilized by detergents sodium dodecyl sulphate and cetrimonium bromide, followed by chloroform isopropanol extraction [13].

### Genotyping of MTBC isolates

Classification of mycobacterial isolates into the main phylogenetic lineages within the MTBC were by TaqMan real-time PCR (TaqMan, Applied Bio systems, USA) using probes targeting lineage-specific SNPs as reported by Stucki et al. [14]. Spoligotyping was performed to define the sub-lineages and strain families within each of the main lineages circulating in Ghana [15]. Spoligotyping patterns were defined according to SITVITWEB database [16] (http://www.pasteur-guadeloupe.fr: 8081/SITVIT_ONLINE). SITVITWEB assigned shared types numbers were used whenever a Spoligotyping pattern was found in the database while families and subfamilies were assigned based on the MIRU-VNTRplus database (http: //www. miru-vntrplus.org) [17].

### Anti-TB Drug Susceptibility Testing

All MTBC isolates were screened for their susceptibility to isoniazid (INH) and Rifampicin (RIF) using the Genotype MTBDR*plus* (Hain lifescience), according to the manufacturer’s protocol [18]. Briefly, drug resistance was expressed as the absence of wild-type band, presence of mutation band or both.

### Data entry, management and analysis

Information from the structured questionnaire was double entered using Microsoft Access and validated to remove duplicates and data entry inconsistencies. Spoligotype patterns were entered in a binary format. A series of univariate and multivariable logistic regression models were fitted to assess the relationship between MTBC lineage (s) (primary independent variable) and host variables. For the MTBDRplus assay, definition of specific drug resistance was based on mutations within the katG gene and inhA promoter for INH and rifampicin resistance determining region of the rpoB gene for RIF. This was captured as either absence of specific wild-type band and/or presence of mutation band corresponding to specific mutations.

## Results

### Patient characteristics

From September 2012 to April 2014, 1,330 smear-positive TB cases recruited from various diagnostic laboratories in the Greater Accra Region and the Northern Region of Ghana were enrolled into the study. We excluded 80 cases from further analysis because no MTBC cultures were obtained from their sputa. A further 30 strains identified as non-tuberculous mycobacteria and 9 strains which produced ambiguous genotyping results were excluded, leaving 1,211 unique MTBC patient isolates for further analyses.

A summary of characteristics of the study population is provided in Table 1. The median age of patients was 39 years (range 3 to 91 years). Thirty-one percent (373/1,211) were females with median age of 33 years; the remaining 838 (69 %) were males with a median age of 36. Only 2.3 % (28/1,211) were children (age < 16 years). Most of the patients (*N* = 1,112; 91.8 %) were recruited from the southern part of Ghana with the remaining 99 (8.2 %) from northern Ghana. Most patients (*N* = 1,160, 95.8 %) were Ghanaians with the remaining 51 (4.2 %) of other West African nationalities. Participants were predominantly of the Akan ethnicity (*N* = 604, 49.9 %), followed by Ga (*N* = 280, 23.1 %), Ewe (*N* = 184, 15.2 %) with the remaining ethnicities (*N* = 143) accounted for 11.8 %. Majority of participants (*N* = 766, 63.3 %) had formal education level of secondary education or lower whilst 346 patients (28.6 %) had no formal education. More than half of our study population (*N* = 591, 48.8 %) were unskilled labourers, 314 skilled (26 %) with the remaining 306 (25.2 %) unemployed. Most of the study participants harbored a high bacterial burden as measured by sputum smear microscopy; 3+ (*N* = 534, 44.1 %), followed by 2+ (*N* = 295, 24.4 %), 1+ (*N* = 266, 22 %) and scanty (*N* = 115, 9.5 %). All 1,211 TB patients consented to HIV testing, and among them, 160 (13.2 %) were HIV sero-positive.

**Table 1:** Characteristics of patients included in the study

Variable	*N* (Total = 1211)	%
*Sex*		
Male	838	69.0
Female	373	31.0
Age category		
yr 08–25	227	18.7
yr 26–40	496	41.0
yr 41–77	488	40.3
*Residency*		
North	99	8.2
South	1112	91.8
*Occupation*		
Unskilled	591	48.8
Skilled	314	26.0
Unemployed	306	25.2
*Nationality*		
Ghanaian	1160	95.8
West Africans	51	4.2
*Income* (*GHC*)		
None	449	37.1
<100	184	15.2
100–500	505	41.7
>500	73	6.0
*Smear positivity*		
Scanty 1–9	115	9.5
+1	266	22.0
+2	295	24.4
+3	534	44.1
*Ethnicity*		
Akan	604	49.9
Ewe	184	15.2
Ga	280	23.1
other	143	11.8
*HIV status*		
Positive	160	13.2
*Education*		
No Education	346	28.6
Primary	134	11.1
Secondary	632	52.2
Tertiary	99	8.2
*Presence of BCG Scar*		
Yes	505	41.7

### The population structure of the MTBC in Ghana

SNP-typing identified 871 (71.9 %) isolates as Lineage 4 (also known as the Euro American lineage), 152 isolates (12.6 %) as Lineage 5, 112 isolates (9.2 %) as Lineage 6, 15 isolates (1.2 %) as Lineage 1, 42 isolates (3.5 %) as Lineage 2, 12 isolates (1 %) as Lineage 3, and 7 (0.6 %) as *M. bovis* (Additional file 1: Table S1). Among the 871 Lineage 4 isolates, spoligotyping revealed that 503/871 (58 %) belonged to the ‘Cameroon family’, the most dominant sub-lineage of Lineage 4 in Ghana, with the most prevalent spoligotype 61 accounting for 349 (40.1 %) isolates. Seventy–three percent of strains isolated from other West African nationals (37/51) belonged to the ‘Cameroon family’.

In addition to the Cameroon family, seven other sub-lineages were identified among Lineage 4 based on spoligotyping; Ghana (*N* = 198/871, 22.7 %), Haarlem (*N* = 83, 9.5 %), Uganda I (*N* = 27, 3.0 %), LAM (*N* = 26, 2.9 %), Uganda II (*N* = 2, 0.2 %), S (*N* = 2 (0.2 %), New_1 (*N* = 1, 0.1 %) and H37Rv_like (*N* = 1, 0.1 %).

Spoligotyping of the 264 MAF (Lineage 5 and 6 combined) isolates revealed 92 distinct spoligotypes patterns. Fifty-two unique patterns (singletons) were observed with the remaining 40 patterns grouped into clusters of 2–26 isolates respectively (Additional file 1: Table S1). Within the 940 MTB isolates, we had 101 patterns, consisting of 60 patterns grouped into clusters of 2–349 isolates respectively, 19 singletons and remaining 11 isolates identified as orphans. We compared the 193 spoligotypes found in this study with those contained in an international spoligotype database, 130 of our spoligotypes were already described in SITVIT database. The other 63 spoligotypes were novel.

### Prevalence of drug resistance among the main MTBC lineages

After excluding the 7 *M. bovis* isolates, all remaining 1,204 isolates were analysed by GenoType® MTBDR*plus usi*ng mutations as specified by manufacturer (Additional file 2: Table S2). Overall, MTBDR*plus* identified 90/1,204 (7.5 %), 7/1,204 (0.6 %) and 21/1,211 (1.7 %) of the isolates as INH-mono-resistant, RIF mono-resistant and MDR, respectively (Table 2). Among our data set, the number of isolates belonging to the Lineages 1–3 was limited. Therefore, for the remaining analyses, we compared Lineage 5 (*N* = 152) and Lineage 6 (*N* = 112) with all the other MTB lineages combined (*N* = 940, Lineages 1–4). Based on univariate analysis, we did not find any association between MDR and Lineage 5 compared to Lineages 1–4 (OR 1.1, 95 % CI: 0.6–2.0), but Lineage 6 was four times less likely to harbour any form of INH resistance (OR = 0.25 95 % CI: 0.05–0.77, Fischer’s Exact test *P* < 0.01). All the RIF mono-resistant isolates (*N* = 7) belonged to Lineage 4.

**Table 2:** Prevalence of drug resistance among the main MTBC lineages

Drug Resistance	Lineage (Number of Isolates)		
	Lineage 4 (940)	Lineage 5 (152)	Lineage 6 (112)
INH^mono^	74 (7.9 %)	14 (7.9 %)	2 (3.6 %)
RIF^mono^	7 (0.7 %)	0 (0.0 %)	0 (0.0 %)
MDR	15 (1.6 %)	6 (3.3 %)	0 (0.0 %)

### MTBC lineage associations with patient characteristics

Further univariate analyses revealed that Lineage 5 was significantly associated with Ewe ethnicity (OR = 3.0, 95 % CI: 1.5–4.7) (Table 3). Similarly, Lineage 6 was significantly associated with HIV co-infection (OR = 2.4, 95 % CI: 1.4–3.9). Both of these associations remained statistically significant after adjusting for age and gender using multivariate logistic modeling, (adjusted odds ratio (adjOR) =2.79, 95 % CI: 1.47–5.29, and adjOR = 2.2, 95 % CI: 1.32–3.7, respectively). No other significant association was found between MTBC lineage and other patient variables, including age, income, sex, the presence of a BCG scar, or the degree of smear positivity.

**Table 3:** Distribution of patient variables across the three main MTBC lineages in Ghana

Risk factor	MAF Lineage 5 (*N* = 152)	MAF Lineage 6 (*N* = 112)	MTB (Lineages 1–4) (*N* = 940)	OR (95 % CI) (Lineage 5 vs. 1–4)	adjOR (95 % CI)^a^ (Lineage 5 vs 1–4)	OR (95 % CI) (Lineage 6 vs 1–4)	adjOR (95 % CI)^a^ (Lineage 6 vs 1–4)
	*N* (%	*N* (%	*N* (%				
*Sex*							
Male	96 (63.2)	73 (65.2)	662 (70.6)	1.3 (0.97–1.98)			
Female	56 (36.8)	39 (34.8)	278 (29.4)				
*Age category*							
yr 08–25	28 (18.0)	17 (15.2)	181 (19.4)	1.42 (0.82–2.64)			
yr 26–40	62 (41.0)	49 (43.8)	383 (40.6)	0.96 (0.4–1.96)			
yr 41–77	62 (41.0)	46 (41.0)	376 (40.0)	1.06 (0.59–1.91)			
*Residency*							
North	8 (5.3)	8 (7.1)	82 (9.0)	0.58 (0.27–1.22)			
South	144 (94.7)	104 (92.2)	858 (91.0)	ref			
*Occupation*							
Skilled	35 (23.0)	24 (21.4)	171 (27.2)	0.9 (0.81–1.0)			
Unskilled	117 (77.0)	88 (78.6)	769 (72.8)	ref			
*Income* (*GHC*)							
None	65 (42.7)	43 (38.4)	260 (47.3)	0.8 (0.62–0.98)^*^			
<100	32 (21.1)	16 (14.3)	136 (14.5)	0.91 (0.76–1.0)			
100–500	51 (33.5)	45 (40.2)	408 (32.3)	ref			
>500	4 (2.6)	8 (7.1)	58 (5.9)	1.3 (0.97–1.98)			
*Smear positivity*							
Scanty 1–9	2 (1.3)	0	80 (9.2)	0.6 (0.45–0.72)^*^			
+1	34 (22.6)	18 (16.1)	199 (22.8)	0.78 (0.65–1.80)			
+2	37 (24.3)	28 (25.0)	213 (24.6)	0.98 (0.42–1.84)			
+3	79 (52.0)	66 (58.9)	352 (40.4)	ref			
*Ethnicity*							
Akan	39 (25.7)	78 (69.7)	482 (51.4)	ref			
Ewe	58 (38.2)	10 (8.9)	116 (12.6)	3.0 (1.5–4.70)	2.79 (1.47–5.29)^**^		
Ga	47 (30.9)	11 (9.8)	221 (22.6)		0.85 (0.43–1.69)		
Other	8 (5.3)	13 (11.6)	118 (13.3)		1.64 (0.53–5.34)		
*HIV status*							
Positive	28 (29.5)	29 (34.9)	96 (18.5)			2.4 (1.4–3.90)^*^	2.2 (1.32–3.70)^*^
*Education*							
No Education	50 (32.9)	40 (35.7)	231 (26.5)	0.83 (0.75–1.0)			
Primary	21 (13.8)	15 (13.4)	91 (10.4)	0.72 (0.65–1.11)			
Secondary	70 (46.1)	47 (42.0)	477 (54.8)	0.96 (0.83–1.30)			
Tertiary	11 (7.2)	10 (8.9)	72 (8.3)				
*BCG Scar*							
Present	64 (42.1)	46 (41.1)	430 (41.4)	1.1 (0.69–1.7)			
*Smoking*							
Yes	25 (16.4)	28 (25.0)	248 (20.6)	0.6 (0.7–1.8)			

^a^All variables with an OR above 1.5 or below 2/3 were included in the multivariable model, *P < 0.05, **P < 0.001, MAF = *Mycobacterium africanum*

*MTB mycobacterium tuberculosis* sensu stricto

## Discussion and conclusions

Our molecular epidemiological analysis revealed that i) MAF remains an important pathogen in Ghana, causing one fifth of all human TB cases in Ghana, ii) TB patients of the Ewe ethnicity were more likely to be infected with Lineage 5, and iii) Lineage 6 isolates were less likely to be INH-resistant and more frequent in TB patients co-infected with HIV.

The observation that MAF remains an important cause of pulmonary TB in Ghana is in contrast to the decline of MAF that has been reported in Cameroon [19]. In that country, MAF might gradually being out-competed by more virulent MTB. In support for this hypothesis, MAF has been associated with reduced virulence in animal models [4], and a longer latency and a slower rate of progression to active disease in humans [9]. The reason for the persistence of MAF in Ghana over time is unclear but might be partially related to our previous finding that MAF is associated with the Ewe ethnic group [12]. Lineage 5, a finding that we replicated in the present study, drove this association. While we were not able to control for HIV infection in our previous study, in the present study, the patient HIV status was available and included in the analysis. Yet despite this potential confounding factor, the association between Lineage 5 and Ewe ethnicity remained significant. Based on these findings, we hypothesize that Lineage 5 is particularly well adapted to infect and cause TB in the Ewe population [12].

This hypothesis follows on an increasing body of evidence indicating that different MTBC genotypes might be adapted to different human populations [20–23]. Moreover, several studies have reported interactions between host and pathogen genotypes in human TB [22–24]. For example, a study in Vietnam found an association between the T597C allele of the Toll-like receptor 2 gene (TLR2) and infection by Lineage 2 [25]. Similarly, studies performed in Ghana have reported human polymorphism in 5-lipoxygenase (ALOX5) [26] and Mannose Binding Lectin (MBL) which gives protection to TB caused by either MAF or MTB [27]. Most recently, a study in South Africa reported on the association between different HLA class I types and disease caused by different MTBC strain families in a South African indigenous population [28]. More studies are needed to confirm the hypothesis that the genetic background of Ewe populations is particularly conducive to infection and disease caused by Lineage 5 and to identify the biological processes involved in this interaction.

Even if the association between Lineage 5 and Ewe ethnicity is contributing to the stable prevalence of MAF in Ghana over time, about half of all MAF cases are due to Lineage 6, which is phylogenetically distinct from Lineage 5 [29]. The findings reported here indicate that Lineage 5 and Lineage 6 also differ phenotypically with respect to their associations with particular patient variables. We found that Lineage 6 was less likely to show any form of INH resistance. This is consistent with reports from Mali that found Lineage 6 less likely to be multidrug-resistant compared to other MTBC lineages [30]. Drug resistance in many bacteria, including MTBC is often associated with a fitness cost [31]. Hence, the fact that Lineage 6 is already attenuated compared to other MTBC lineages [9, 32] might limit its potential for drug resistance acquisition. In support of this idea, Lineage 6 has been associated with *inhA* promoter mutations conferring low levels of resistance to isoniazid that have no known impact on bacterial fitness [33]. In contrast, most other MTBC strains tend to harbour mutations in *katG* conferring high levels of resistance, some of which are associated with a high fitness cost [34].

The relative attenuation of Lineage 6 has led to the hypothesis that this lineage might be an opportunistic pathogen [35]. This view is supported by the findings reported here, as well as previous work by others [9] showing an association between Lineage 6 and HIV co-infection [34]. A previous study from Ghana found no such association, perhaps reflecting differences in study design [36]. Moreover, de Jong et al. reported that Lineage 6 was significantly more frequent in elderly TB patients, consistent with both slower progression to active disease [37] and the role of age-related immunosuppression [34]. Recent advances in phylogenomics of the MTBC show that even though Lineage 6 has primarily been isolated from humans, it is phylogenetically most closely related to several animal-adapted variants of the MTBC [24] including the recently discovered chimpanzee bacillus [38] as well as *M. suricattae* and *M. mungi* which cause TB-like disease in meerkats and banded mangooses, respectively [39]. The potential for an unknown animal reservoir for Lineage 6 has been discussed for some time but so far, none has been discovered [9]. Moreover, unlike other animal-adapted members of the MTBC that rarely cause TB in humans and cannot maintain a full life cycle in human populations [40], Lineage 6 transmits readily among humans [9]. So far, the role of HIV in driving Lineage 6 remains unclear. Even though HIV had a dramatic impact on the TB epidemic in Sub-Saharan Africa, Lineage 6 remains restricted to the Western part of the continent, which shows much lower rates of HIV infection compared to Southern Africa. Hence, other factors are likely to determine to epidemiology of Lineage 6.

In conclusion, our study confirms that MAF remains an important cause of human TB in Ghana. Our findings also emphasize the need to differentiate between Lineage 5 and 6, and show that the differences between these lineages are relevant for understanding the phylogeography of MAF; while human genetic factors may drive Lineage 5, unknown environmental factors seem to influence the epidemiology of Lineage 6. More generally, these results also highlight the current problems with the species nomenclature in the MTBC [39]. Indeed, the phylogenetic, phenotypic, and ecological differences between Lineage 5 and Lineage 6 argue against them being collectively referred to as MAF, as opposed to just separate lineages within MTB. Finally, given the current efforts in the development of new TB vaccines, our findings also highlight the need to consider the MTBC variability in the development of new tools and strategies to control TB especially in area where Lineages 5 and 6 are prevalent.

## Abbreviations

AFB, acid fast bacilli; AIDS, acquired immune deficiency syndrome; BCG, bacillus calmette-guerin; DNA, deoxyribonucleic acid; EKBB, ethikkommission beider basel; HIV, human immunodeficiency virus infection; INH, isoniazid; MAF, mycobacterium africanum; MBL, mannose binding lectin; MDR, multi drug resistance; MTB, mycobacterium tuberculosis; MTBC, mycobacterium tuberculosis complex; NMIMR, noguchi memorial institute for medical research; NTP, national tuberculosis programme; RIF, rifampicin; TB, tuberculosis; TLR, toll like receptor

## Additional files

Additional file 1: Table S1. Interpretation of MTBDR*plus* results with specific mutations. (DOC 43 kb) Additional file 2: Table S2. Genotyping profile of 1211 MTBC isolates from Ghana. (DOC 577 kb)
