# Supplementary material for: Molecular epidemiology of Mycobacterium africanum in Ghana
Source: BMC Infect Dis. 2016 Aug 9;16:385. doi: 10.1186/s12879-016-1725-6 (PMC4977717; doi:10.1186/s12879-016-1725-6)
Supplement: Additional file 1: Table S1. — Interpretation of MTBDRplus results with specific mutations. (DOC 43 kb) [file 12879_2016_1725_MOESM1_ESM.doc]

**Additional file 1: Table S1. Interpretation of MTBDR*plus*** results with specific mutations

| Locus | Failing Wild-Type bands | Codons involved | Developing Mutation bands | Specific Mutation |
| --- | --- | --- | --- | --- |
| RRDR of *ropB* | *rpoB* WT1 | 424-428 |  | F424L, T427A,S428T |
|  | *rpoB* WT2 | 429-432 |  | L430P* |
|  | *rpoB* WT2/3 | 429-436 |  | Q432L*,Q432P, del433-435 |
|  | *rpoB* WT3/4 | 432-438 | *rpoB* Mut1 | D435V,D435Y,del434 |
|  | *rpoB* WT4/5 | 435-441 |  | Del437*,N437I |
|  | *rpoB* WT5/6 | 437-444 |  | S441L,S441Q |
|  | *rpoB* WT7 | 445-448 | *ropB* Mut2A, *rpoB* Mut2B | H445Y,H445D,H445R,H445P*,H445Q*  H445N,H445L,H445S,H445C |
|  | *rpoB* WT8 | 449-452 | *rpoB* Mut3 | S450L,S450P,S450Q*,S450W,L452P |
| *katG* | *katG* WT | 315 | *katG* Mut1 | S315T1 |
|  |  |  | *katG* Mut2 | S315T2 |
| *inhApro* | *inhA* WT1 | -15 | *inhA* Mut1 | C15T |
|  |  | -16 | *inhA* Mut2 | A16G |
|  | *inhA* WT2 | -8 | *inhA* Mut3A | T8C |
|  |  |  | *inhA* Mut3B | T8A |

NB: * This rare mutation has been only possible *in silico* but has not been yet detected *in vitro* or *in vivo*
