# Supplementary material for: Molecular epidemiology of Mycobacterium africanum in Ghana
Source: BMC Infect Dis. 2016 Aug 9;16:385. doi: 10.1186/s12879-016-1725-6 (PMC4977717; doi:10.1186/s12879-016-1725-6)
Supplement: Additional file 2: Table S2. — Genotyping profile of 1211 MTBC isolates from Ghana. (DOC 577 kb) [file 12879_2016_1725_MOESM2_ESM.doc]

| **Additilnal file 2: Table S2. Genotyping profile of 1211 MTBC isolates from Ghana** | | | | | | |
| --- | --- | --- | --- | --- | --- | --- |
| **Specie** | **SNP** | **Spoligotyping profile** | **Sub**  **lineage** | **SIT** | **No** | **%** |
| MTBss | L1 | 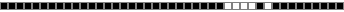 | EAI | 236 | 1 | 0.08 |
| MTBss | L1 | 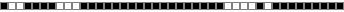 | EAI | 340 | 3 | 0.24 |
| MTBss | L1 | 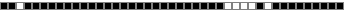 | EAI | 342 | 7 | 0.60 |
| MTBss | L1 | 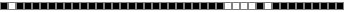 | EAI | 380 | 1 | 0.08 |
| MTBss | L1 | 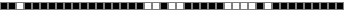 | EAI | Orphan | 1 | 0.08 |
| MTBss | L1 | 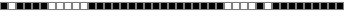 | EAI |  | 2 | 0.16 |
| MTBss | L2 | 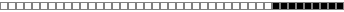 | Beijing | 1 | 36 | 3.00 |
| MTBss | L2 | 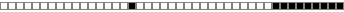 | Beijing | 1184 | 3 | 0.24 |
| MTBss | L2 | 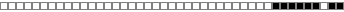 | Beijing | 941 | 1 | 0.08 |
| MTBss | L2 | 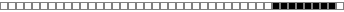 | Beijing |  | 2 | 0.17 |
| MTBss | L3 | 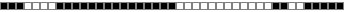 | Dehli_CAS | 25 | 1 | 0.08 |
| MTBss | L3 | 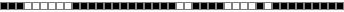 | Dehli_CAS | 129 | 1 | 0.08 |
| MTBss | L3 | 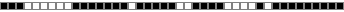 | Dehli_CAS | 702 | 1 | 0.08 |
| MTBss | L3 | 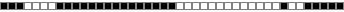 | Dehli_CAS | 1199 | 2 | 0.17 |
| MTBss | L3 | 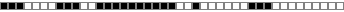 | Dehli_CAS |  | 2 | 0.17 |
| MTBss | L3 | 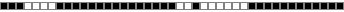 | Dehli_CAS |  | 3 | 0.24 |
| MTBss | L3 | 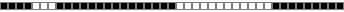 | Dehli_CAS | Orphan | 1 | 0.08 |
| MTBss | L3 | 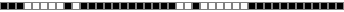 | Dehli_CAS | Orphan | 1 | 0.08 |
| MTBss | L4 | 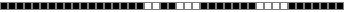 | Cameroon | 57 | 5 | 0.41 |
| MTBss | L4 | 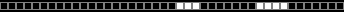 | Cameroon | 61 | 349 | 29.0 |
| MTBss | L4 | 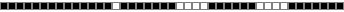 | Cameroon | 114 | 1 | 0.08 |
| MTBss | L4 | 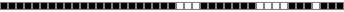 | Cameroon | 403 | 2 | 0.17 |
| MTBss | L4 | 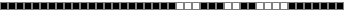 | Cameroon | 772 | 39 | 3.22 |
| MTBss | L4 | 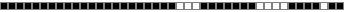 | Cameroon | 838 | 12 | 1.00 |
| MTBss | L4 | 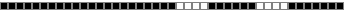 | Cameroon | 1141 | 8 | 0.67 |
| MTBss | L4 | 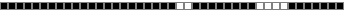 | Cameroon | 1580 | 1 | 0.08 |
| MTBss | L4 | 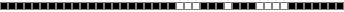 | Cameroon |  | 10 | 0.82 |
| MTBss | L4 | 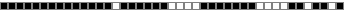 | Cameroon |  | 15 | 1.25 |
| MTBss | L4 | 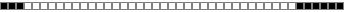 | Cameroon |  | 5 | 0.41 |
| MTBss | L4 | 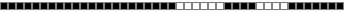 | Cameroon |  | 15 | 1.25 |
| MTBss | L4 | 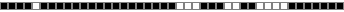 | Cameroon |  | 7 | 0.60 |
| MTBss | L4 | 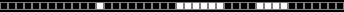 | Cameroon |  | 15 | 1.25 |
| MTBss | L4 | 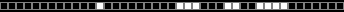 | Cameroon |  | 7 | 0.60 |
| MTBss | L4 | 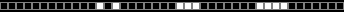 | Cameroon |  | 4 | 0.33 |
| MTBss | L4 | 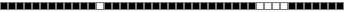 | Ghana | 37 | 1 | 0.08 |
| MTBss | L4 | 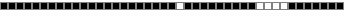 | Ghana | 44 | 1 | 0.08 |
| MTBss | L4 | 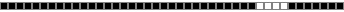 | Ghana | 53 | 138 | 11.40 |
| MTBss | L4 | 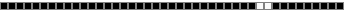 | Ghana | 54 | 3 | 0.24 |
| MTBss | L4 | 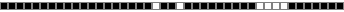 | Ghana | 58 | 1 | 0.08 |

| Specie | SNP | Spoligotyping profile | Sub lineage | SIT | No | % |
| --- | --- | --- | --- | --- | --- | --- |
| MTBss | L4 | 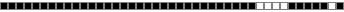 | Ghana | 278 | 1 | 0.08 |
| MTBss | L4 | 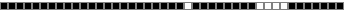 | Ghana | 373 | 1 | 0.08 |
| MTBss | L4 | 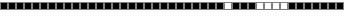 | Ghana | 462 | 3 | 0.24 |
| MTBss | L4 | 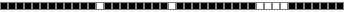 | Ghana | 504 | 25 | 2.10 |
| MTBss | L4 | 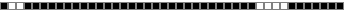 | Ghana | 804 | 1 | 0.08 |
| MTBss | L4 | 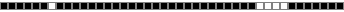 | Ghana | 926 | 1 | 0.08 |
| MTBss | L4 | 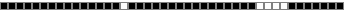 | Ghana | 1105 | 1 | 0.08 |
| MTBss | L4 | 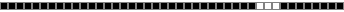 | Ghana | 1196 | 2 | 0.17 |
| MTBss | L4 | 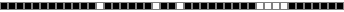 | Ghana | 1227 | 2 | 0.17 |
| MTBss | L4 | 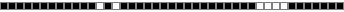 | Ghana | 1547 | 1 | 0.08 |
| MTBss | L4 | 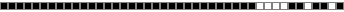 | Ghana |  | 4 | 0.33 |
| MTBss | L4 | 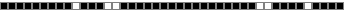 | Ghana |  | 3 | 0.24 |
| MTBss | L4 | 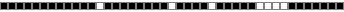 | Ghana | orphan | 1 | 0.08 |
| MTBss | L4 | 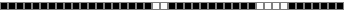 | H37Rv | orphan | 1 | 0.08 |
| MTBss | L4 | 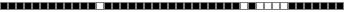 | Haarlem | 36 | 1 | 0.08 |
| MTBss | L4 | 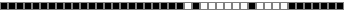 | Haarlem | 45 | 3 | 0.24 |
| MTBss | L4 | 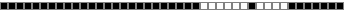 | Haarlem | 47 | 1 | 0.08 |
| MTBss | L4 | 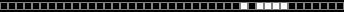 | Haarlem | 50 | 26 | 2.14 |
| MTBss | L4 | 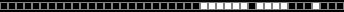 | Haarlem | 62 | 1 | 0.08 |
| MTBss | L4 | 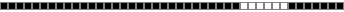 | Haarlem | 124 | 1 | 0.08 |
| MTBss | L4 | 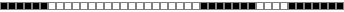 | Haarlem | 144 | 2 | 0.17 |
| MTBss | L4 | 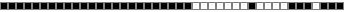 | Haarlem | 316 | 8 | 0.67 |
| MTBss | L4 | 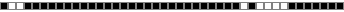 | Haarlem | 655 | 11 | 0.90 |
| MTBss | L4 | 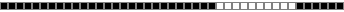 | Haarlem | 775 | 3 | 0.24 |
| MTBss | L4 | 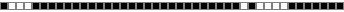 | Haarlem | 1159 | 1 | 0.08 |
| MTBss | L4 | 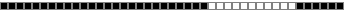 | Haarlem | 1498 | 11 | 0.90 |
| MTBss | L4 | 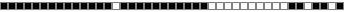 | Haarlem |  | 4 | 0.33 |
| MTBss | L4 | 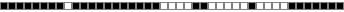 | Haarlem |  | 3 | 0.24 |
| MTBss | L4 | 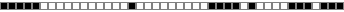 | Haarlem |  | 3 | 0.24 |
| MTBss | L4 | 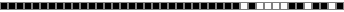 | Haarlem |  | 3 | 0.24 |
| MTBss | L4 | 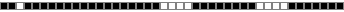 | LAM | 20 | 1 | 0.08 |
| MTBss | L4 | 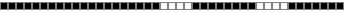 | LAM | 42 | 13 | 1.10 |
| MTBss | L4 | 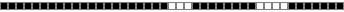 | LAM | 535 | 2 | 0.17 |
| MTBss | L4 | 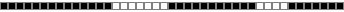 | LAM | 765 | 1 | 0.08 |
| MTBss | L4 | 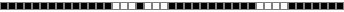 | LAM |  | 2 | 0.17 |
| MTBss | L4 | 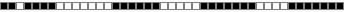 | LAM | orphan | 1 | 0.08 |
| MTBss | L4 | 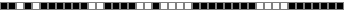 | LAM |  | 2 | 0.17 |
| MTBss | L4 | 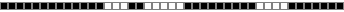 | LAM |  | 2 | 0.17 |
| MTBss | L4 | 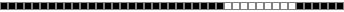 | NEW | orphan | 1 | 0.08 |
| MTBss | L4 | 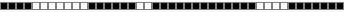 | S |  | 2 | 0.17 |
| MTBss | L4 | 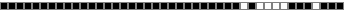 | Uganda I | 49 | 2 | 0.17 |
| MTBss | L4 | 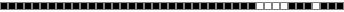 | Uganda I | 52 | 2 | 0.17 |
| Specie | SNP | Spoligotyping profile | Sub lineage | SIT | No | % |
| MTBss | L4 | 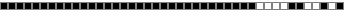 | Uganda I | 78 | 1 | 0.08 |
| MTBss | L4 | 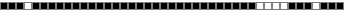 | Uganda I | 848 | 1 | 0.08 |
| MTBss | L4 | 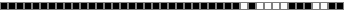 | Uganda I | 524 | 1 | 0.08 |
| MTBss | L4 | 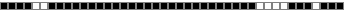 | Uganda I | 712 | 1 | 0.08 |
| MTBss | L4 | 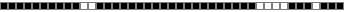 | Uganda I | 1056 | 1 | 0.08 |
| MTBss | L4 | 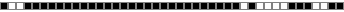 | Uganda I |  | 10 | 0.82 |
| MTBss | L4 | 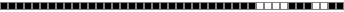 | Uganda I |  | 3 | 0.24 |
| MTBss | L4 | 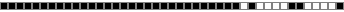 | Uganda I |  | 4 | 0.33 |
| MTBss | L4 | 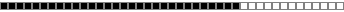 | Uganda II | 237 | 2 | 0.17 |
| MTBss | L4 | 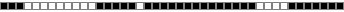 | Uganda II | 92 | 11 | 0.90 |
| MTBss | L4 | 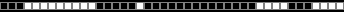 | Uganda II | 200 | 5 | 0.41 |
| MTBss | L4 | 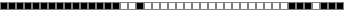 | Uganda II | 1178 | 4 | 0.33 |
| MTBss | L4 | 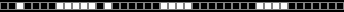 | Uganda II |  | 5 | 0.41 |
| MTBss | L4 | 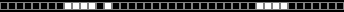 | Uganda II | orphan | 1 | 0.08 |
| MTBss | L4 | 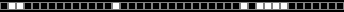 | Uganda II |  | 6 | 0.50 |
| MTBss | L4 | 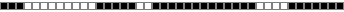 | Uganda II |  | 2 | 0.17 |
| MTBss | L4 | 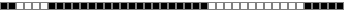 | Uganda II |  | 4 | 0.33 |
| MTBss | L4 | 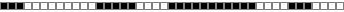 | Uganda II | orphan | 1 | 0.08 |
| MTBss | L4 | 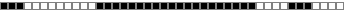 | Uganda II |  | 3 | 0.24 |
| MTBss | L4 |  | Uganda II | orphan | 1 | 0.08 |
| *Mafric* | L5 |  | West Africa I | 319 | 26 | 2.14 |
| *Mafric* | L5 |  | West Africa I | 320 | 3 | 0.24 |
| *Mafric* | L5 |  | West Africa I | 330 | 4 | 0.33 |
| *Mafric* | L5 |  | West Africa I | 331 | 26 | 2.14 |
| *Mafric* | L5 |  | West Africa I | 438 | 5 | 0.41 |
| *Mafric* | L5 |  | West Africa I | 1592 | 2 | 0.17 |
| *Mafric* | L5 |  | West Africa I |  | 15 | 1.24 |
| *Mafric* | L5 |  | West Africa I | orphan | 1 | 0.08 |
| *Mafric* | L5 |  | West Africa I | orphan | 1 | 0.08 |
| *Mafric* | L5 |  | West Africa I | orphan | 1 | 0.08 |
| *Mafric* | L5 |  | West Africa I | orphan | 1 | 0.08 |
| *Mafric* | L5 |  | West Africa I |  | 3 | 0.24 |
| *Mafric* | L5 |  | West Africa I | orphan | 1 | 0.08 |
| *Mafric* | L5 |  | West Africa I |  | 2 | 0.17 |
| *Mafric* | L5 |  | West Africa I |  | 2 | 0.17 |
| *Mafric* | L5 |  | West Africa I | orphan | 1 | 0.08 |

| Specie | SNP | Spoligotyping profile | Sub lineage | SIT | No | % |
| --- | --- | --- | --- | --- | --- | --- |
| *Mafric* | L5 |  | West Africa I | orphan | 1 | 0.08 |
| *Mafric* | L5 |  | West Africa I | orphan | 1 | 0.08 |
| *Mafric* | L5 |  | West Africa I |  | 2 | 0.17 |
| *Mafric* | L5 |  | West Africa I | orphan | 1 | 0.08 |
| *Mafric* | L5 |  | West Africa I | orphan | 1 | 0.08 |
| *Mafric* | L5 |  | West Africa I | orphan | 1 | 0.08 |
| *Mafric* | L5 |  | West Africa I | orphan | 1 | 0.08 |
| *Mafric* | L5 |  | West Africa I |  | 3 | 0.24 |
| *Mafric* | L5 |  | West Africa I | orphan | 1 | 0.08 |
| *Mafric* | L5 |  | West Africa I | orphan | 1 | 0.08 |
| *Mafric* | L5 |  | West Africa I |  | 2 | 0.17 |
| *Mafric* | L5 |  | West Africa I |  | 2 | 0.17 |
| *Mafric* | L5 |  | West Africa I |  | 2 | 0.17 |
| *Mafric* | L5 |  | West Africa I |  | 2 | 0.17 |
| *Mafric* | L5 |  | West Africa I | orphan | 1 | 0.08 |
| *Mafric* | L5 |  | West Africa I | orphan | 1 | 0.08 |
| *Mafric* | L5 |  | West Africa I | orphan | 1 | 0.08 |
| *Mafric* | L5 |  | West Africa I |  | 5 | 0.41 |
| *Mafric* | L5 |  | West Africa I |  | 2 | 0.17 |
| *Mafric* | L5 |  | West Africa I | orphan | 1 | 0.08 |
| *Mafric* | L5 |  | West Africa I | orphan | 1 | 0.08 |
| *Mafric* | L5 |  | West Africa I |  | 2 | 0.17 |
| *Mafric* | L5 |  | West Africa I | orphan | 1 | 0.08 |
| *Mafric* | L5 |  | West Africa I | orphan | 1 | 0.08 |
| *Mafric* | L5 |  | West Africa I | orphan | 1 | 0.08 |
| *Mafric* | L5 |  | West Africa I | orphan | 1 | 0.08 |
| *Mafric* | L5 |  | West Africa I |  | 2 | 0.17 |

| Specie | SNP | Spoligotyping profile | Sub lineage | SIT | No | % |
| --- | --- | --- | --- | --- | --- | --- |
| Mafric | L5 |  | West Africa I | orphan | 1 | 0.08 |
| Mafric | L5 |  | West Africa I |  | 2 | 0.17 |
| Mafric | L5 |  | West Africa I | orphan | 1 | 0.08 |
| Mafric | L5 |  | West Africa I |  | 4 | 0.33 |
| Mafric | L5 |  | West Africa I | orphan | 1 | 0.08 |
| Mafric | L5 |  | West Africa I | orphan | 1 | 0.08 |
| Mafric | L5 |  | West Africa I | orphan | 1 | 0.08 |
| Mafric | L5 |  | West Africa I | orphan | 1 | 0.08 |
| Mafric | L5 |  | West Africa I | orphan | 1 | 0.08 |
| Mafric | L5 |  | West Africa I | orphan | 1 | 0.08 |
| Mafric | L5 |  | West Africa I | orphan | 1 | 0.08 |
| Mafric | L5 |  | West Africa I | orphan | 1 | 0.08 |
| Mafric | L6 |  | West Africa II | 181 | 23 | 1.90 |
| Mafric | L6 |  | West Africa II | 326 | 24 | 2.00 |
| Mafric | L6 |  | West Africa II | 1200 | 1 | 0.08 |
| Mafric | L6 |  | West Africa II | 1867 | 1 | 0.08 |
| Mafric | L6 |  | West Africa II |  | 4 | 0.33 |
| Mafric | L6 |  | West Africa II |  | 4 | 0.33 |
| Mafric | L6 |  | West Africa II | orphan | 1 | 0.08 |
| Mafric | L6 |  | West Africa II | orphan | 1 | 0.08 |
| Mafric | L6 |  | West Africa II | orphan | 1 | 0.08 |
| Mafric | L6 |  | West Africa II |  | 19 | 1.57 |
| Mafric | L6 |  | West Africa II |  | 3 | 0.24 |
| Mafric | L6 |  | West Africa II |  | 3 | 0.24 |
| Mafric | L6 |  | West Africa II | orphan | 1 | 0.08 |
| Mafric | L6 |  | West Africa II |  | 3 | 0.24 |
| Mafric | L6 |  | West Africa II |  | 3 | 0.24 |

| Specie | SNP | Spoligotyping profile | Sub lineage | SIT | No | % |
| --- | --- | --- | --- | --- | --- | --- |
| Mafric | L6 |  | West Africa II | orphan | 1 | 0.08 |
| Mafric | L6 |  | West Africa II |  | 5 | 0.41 |
| Mafric | L6 |  | West Africa II | orphan | 1 | 0.08 |
| Mafric | L6 |  | West Africa II | orphan | 1 | 0.08 |
| Mafric | L6 |  | West Africa II |  | 4 | 0.33 |
| Mafric | L6 |  | West Africa II | orphan | 1 | 0.08 |
| Mafric | L6 |  | West Africa II | orphan | 1 | 0.08 |
| Mafric | L6 |  | West Africa II | orphan | 1 | 0.08 |
| Mafric | L6 |  | West Africa II | orphan | 1 | 0.08 |
| Mafric | L6 |  | West Africa II | orphan | 1 | 0.08 |
| Mafric | L6 |  | West Africa II | orphan | 1 | 0.08 |
| Mafric | L6 |  | West Africa II | orphan | 1 | 0.08 |
| Mafric | L6 |  | West Africa II | orphan | 1 | 0.08 |
| M. bovis |  |  | BOVIS 1_BCG | 482 | 1 | 0.08 |
| M. bovis |  |  | BOVIS 1 | 1037 | 2 | 0.17 |
| M. bovis |  |  | BOVIS | orphan | 1 | 0.08 |
| M. bovis |  |  | BOVIS | orphan | 1 | 0.08 |
| M. bovis |  |  | BOVIS | orphan | 1 | 0.08 |
| M. bovis |  |  | BOVIS | orphan | 1 | 0.08 |
